# Supplementary material for: Efficient simulation of non-Markovian dynamics on complex networks
Source: PLoS One. 2020 Oct 30;15(10):e0241394. doi: 10.1371/journal.pone.0241394 (PMC7598478; doi:10.1371/journal.pone.0241394)
Supplement: Attachment — Submitted filename: Response_to_Reviewers.pdf [file pone.0241394.s001.pdf]

Response to Reviewers

Deare Reviewers,

We thank you for your constructive and detailed reviews, which contain a lot of valuable suggestions to improve the paper's readability and better explain certain points.

Before we address the reviews individually, we shortly summarize our changes to the manuscript. Generally speaking, we noticed a somewhat contradictory direction in the reviews. Particularly, reviewer 1 seems to be in favor of more extensive explanations while reviewer 3 seems to prefer a shorter and denser manuscript. We tried our best to satisfy both and make the explanations clearer and more concise. The largest difference is the updated "Case studies"-section where, following the reviewers' requests, we added performance results on competing algorithms. Therefore, we changed one of the experiments (the voter model) to make it suitable for an LGA-type approach. We also extended and partially re-wrote the correctness-section and the part where we explain how time-delays are generated.

Next, we answer to specific concerns raised by the reviewers. For clarity, small issues like typos/formulations are updated without explicitly mentioning them here or highlighting them in the revised manuscript.

Review 1

> *I understand that this work is is an extension of [26] in terms of theoretical analysis, at the same time it feels that it needs to be more clear for those who did not read that reference.*

There is a paragraph ("Contribution") explaining the relationship to [26]. In addition, we now added the reference to the Section on "Previous simulation approaches".

> *I would suggest to include a schme or a diagram*
> *to Network dynamics description on page 10. It would clarify to readers*
> *about what is the role of \psi and \phi functions.*

Given the other conflicting reviews and that we already provide two very detailed examples ("Standard Markovian SIS model" and "Complex cascade model") of Psi and Phi, we decided to not include an additional figure.

it is not clear whether they refer to a network to be fully connected component,

The network is always strongly connected. We added: "*We assume that G is strongly connected. That is, each node is reachable from all other nodes.*" to the model description.

in conclussions I would add eleborations on comparing methods to some other methods when it is performing comparably well

We hope to satisfy this request with the updated (extended) numerical evaluations and the extended discussion about the relation to other methods at the beginning of the "Case Studies"-Section.

from 164 line authors describe delay generation, i would again suggest to give more links to other

We added additional references.

it would be useful to give readers some guidelines in which cases the method performs well and in which it is not as effective (when the update the instantaneous rates of the whole neighborhood in each simulation step would drastically decrease optimality to use other algorithms)

In the subsection "Limitations, we discuss the circumstances under which we expect our method to perform comparably poor.

Review 2

Concerning the case studies, it would be interesting to test the performances with existing algorithms and to see that the results obtained are similar (in the statistical sense) to results obtained with other algorithms.
This would also add to this manuscript compared to the already published paper in the complex networks conference.

We added performance results of comparable methods to the case studies. However, the methods are still somewhat different with regard to their objective (we focus on the network-aspect, not on sampling inter-event times in general). Hence, we also added a short discussion about some considerations and limitations of the comparison at the beginning of Section "Case Studies".

More precisely, in the part about generating time-delays, the validity of the method of integration (Fig. 1 and eq. 1) should be demonstrated. Why is t_v distributed according

to the correct distribution? It may be obvious to the authors, but a small justification would make it clearer to the readers. References should also be provided. Also, Fig. 1d should be clearly explained in the main text and in the caption. References for the rejection-based method should also be provided.

We added an explanation (including a proof-sketch) and a reference. We also explicitly referred to Fig. 1d in the text.

Reasoning in term of intensity instead of PDF is not intuitive for most readers, given that the authors chose to submit to a wide audience journal, it would be nice if they tried to help the readers to understand those concepts.
For example, when they say that if the integral of the intensity is finite, the process might not fire at all with positive probability, this is not obvious for me. Could they give an example or provide a reference?

We are aware of this problem and do our best in the paper to make the concept intuitive. To ease the understanding, we extended the explanation in the Section on “Generating time-delays” and in the Section “Intensities and inter-event times”. We also added a reference to the specific claim.

Another problem is that they never actually clearly explain why, with their method, the rates of the neighbors of a firing agent do not need to be updated. This should be explained in the introduction and in the description of the method.

We explain this at the beginning of the Section “Our method” and in an example in the Subsection “Rate over-approximation”. We re-formulated this example to emphasize its purpose. We also added/extended a high-level explanation at the end of Section “Introduction". In Section “Correctness”, we discuss this issue now in more detail and we revised and extended the correctness proof to ease its understanding.

About the case studies, as said above, when proposing a new simulation methods it sounds normal to show how it compares to existing methods (as long as the codes for these methods is easily available).
Could the authors find a case study where LGA, nMGA and RED could be compared?

We re-implemented nMGA and LGA. Hence we added results on the performance of nMGA for all case studies. Moreover, we changed the voter model such that it becomes feasible for LGA and proposed an LGA-type baseline. We note, however, that a direct comparison is difficult because there are different objectives associated with the related methods. That is, nMGA and LGA aim at sampling the inter-event times efficiently, while we are concerned with reducing the number of updating steps in general. We added a short discussion about this to the manuscript.

Also, it would be nice to see that the (ensemble average of the) results of the simulations agree.

As a consequence of the correctness of our method (and of the baseline) they naturally do. In the revised version, we now emphasize more strongly that our method generates statistically exact trajectories.
The nMGA results converge to the true results with increasing network size. However, we did not add this because the analysis of the approximation error of nMGA is beyond the scope of our manuscript.

Review 3

I think that the manuscript could be much more concise allowing to grab the important ideas more quickly. The number of didactic examples is exaggerated and reading not efficient.

For this, we got conflicting suggestions from the reviewers. Given the two other reviews, we decided to keep the didactic examples (also in the light that the journal addresses a rather broad research community).

Another serious issue is that only CPU times with respect to different methods were presented but no results showing the accuracy of the optimized strategies were discussed. I think that it is a bad balance of content where too much space is devoted to pedagogic aims in detriment to discussion and results on the accuracy.

We do not numerically investigate accuracy because we do not present an approximation method but a method that generates statistically exact trajectories. We now emphasize this more strongly in the manuscript in several (sub-)sections (introduction, correctness, and case studies).

Kind regards,
Gerrit Großmann
